# Weighted Copula Entropy for Structural Pruning in Long-Tailed Autonomous Driving Object Detection

**DOI:** 10.3390/e28030336

**Published:** 2026-03-17

**Authors:** Yue Zhou, Jihui Ma, Honghui Dong

**Affiliations:** 1 School of Traffic and Transportation, Beijing Jiaotong University, No. 3 Shangyuancun, Haidian District, Beijing 100044, China; 18114027@bjtu.edu.cn (Y.Z.); jhma@bjtu.edu.cn (J.M.); 2 State Key Laboratory of Advanced Rail Autonomous Operation, Beijing Jiaotong University, No. 3 Shangyuancun, Haidian District, Beijing 100044, China; 3 The Beijing Research Center of Urban Traffic Information Intelligent Sensing and Service Technologies, Beijing Jiaotong University, No. 3 Shangyuancun, Haidian District, Beijing 100044, China

**Keywords:** autonomous driving, structural pruning, weighted copula entropy, long-tailed traffic scenarios, elastic net regularization

## Abstract

In autonomous driving, deep convolutional neural networks face a core conflict between computational efficiency and safety-critical robustness on resource-constrained onboard computing units. Dominant structural pruning, based on weight magnitude or geometric statistics, fails in long-tailed traffic scenarios by equating parameter magnitude with feature importance and pruning critical filters in the tail classes. To address this, we propose a structural pruning framework that evaluates the semantic utility of features using weighted copula entropy rather than relying solely on their magnitude. Our novel approach integrates Elastic Net regularization for inducing sparsity and weighted copula entropy for unbiased information-theoretic feature selection. By incorporating inverse class frequency weighting into empirical Copula estimation, we decouple feature relevance from sample abundance, ensuring the preservation of rare-class discriminators based on their information content rather than occurrence frequency. Furthermore, this metric is embedded into an enhanced max-relevance and min-redundancy algorithm to eliminate semantic redundancy while maintaining representational diversity. Extensive experiments on the BDD100K dataset with YOLOv5l and YOLOv8l architectures demonstrate that, at a 50% pruning rate, the proposed method reduces FLOPs and parameters by nearly 50%, with only 0.09% mAP@0.5 loss for YOLOv5l and 0.14% mAP@0.5 loss for YOLOv8l, while significantly improving the mAP of the extreme tail class Train from 0% to 3.84% and 2.76% to 5.12%, respectively. It achieves a more favorable trade-off between detection accuracy and computational efficiency than mainstream pruning approaches. This work provides a lightweight scheme for autonomous driving perception models and a new information-theoretic perspective for structured network pruning.

## 1. Introduction

Intelligent Vehicles (IVs) and Advanced Driver Assistance Systems (ADAS) represent a paradigm shift in transportation, promising to drastically reduce accident rates, optimize traffic flow, and liberate human attention [1]. Central to the autonomy of these systems is the perception module—the sensory cortex of the vehicle—which is tasked with constructing a real-time, semantic understanding of the surrounding environment from high-dimensional sensor data [2]. Among the suite of available sensors, visual cameras are indispensable due to their rich texture information, high resolution, and relatively low cost compared to LiDAR or radar systems [3].

The ascendancy of deep learning, particularly Deep Convolutional Neural Networks (DCNNs), has revolutionized computer vision, enabling perception systems to detect and classify traffic participants with unprecedented accuracy [4]. Architectures such as the YOLO (You Only Look Once) series have become the standard-bearers for real-time object detection [5,6,7]. However, the performance gains of these models have come at the cost of exponential growth in model complexity. State-of-the-Art detectors often contain tens of millions of parameters and require hundreds of Giga-Floating Point Operations (GFLOPs) per inference pass [8].

This computational burden presents a fundamental bottleneck for deployment. Unlike the controlled environments of data centers, the inference environment of an intelligent vehicle is heavily constrained by Onboard Computing Units (OCUs), which must operate within strict envelopes of power consumption and thermal dissipation [9]. In safety-critical scenarios like highway driving, high latency is unacceptable; a delay of mere milliseconds can translate to meters of braking distance, potentially distinguishing between a near-miss and a catastrophic collision [1]. Thus, there is an urgent need to compress these sophisticated deep-learning models into lightweight forms while preserving their perceptual fidelity.

Among the spectrum of compression strategies, network pruning, specifically structured pruning, has garnered significant attention. By physically removing redundant channels or filters, it delivers tangible acceleration without necessitating the specialized hardware support often required by unstructured sparsity [10]. Conventional pruning criteria have predominantly relied on weight magnitude, geometric medians, or the statistical properties of feature maps to assess filter importance [11]. However, a critical limitation of these established methods emerges when applied to the open-world scenarios of autonomous driving: the inherent long-tailed distribution characteristic of traffic data. In real-world datasets like BDD100K, majority classes dominate the sample space, while safety-critical minority classes are severely underrepresented [12].

In this paper, we propose a novel structural pruning framework that evaluates the semantic utility of features based on information theory rather than physical magnitude. Compared with existing importance-based methods that rely on the L1-norm or weight absolute values, we analyze the statistical dependence between latent features and target semantic classes to identify filters with high information density. Specifically, we propose Weighted Copula Entropy (WCE) as a robust statistical measure and integrate it into the Max-Relevance Min-Redundancy (mRMR) selection process. By further incorporating Elastic Net regularization, our method effectively decouples feature relevance from the dominance of frequent samples. By incorporating inverse class frequency weighting into empirical copula estimation, our method decouples feature relevance from sample abundance. This mechanism ensures that the preservation of discriminators is driven by their intrinsic information content regarding the target classes rather than their occurrence frequency in the training set. Extensive experiments on the BDD100K dataset demonstrate that our approach effectively balances model complexity and detection performance. The main contributions of this paper are summarized as follows:

1. We propose a novel information-theoretic channel pruning framework tailored for autonomous driving scenarios. Unlike conventional methods that rely on physical magnitude, our approach evaluates the semantic utility of features, capturing non-linear dependencies between latent representations and target categories.
2. We formulate a statistically selection strategy combining WCE with the mRMR principle and Elastic Net regularization. This statistically rigorous metric strictly induces structural sparsity and decouples feature relevance from sample abundance, ensuring robust feature selection even for minority classes.
3. Extensive experiments on the large-scale BDD100K dataset demonstrate that our framework achieves a superior trade-off between efficiency and accuracy. Notably, it significantly enhances the detection performance of extreme tail classes while effectively reducing computational complexity.

Our work effectively bridges the critical gap between lightweight model design and long-tailed object detection in autonomous driving, providing a practical solution for deploying efficient perception systems in open-world environments.

The remainder of this paper is organized as follows: Section 2 reviews related work on structural pruning and information-theoretic feature selection. Section 3 details the proposed Weighted Copula Entropy (WCE) pruning framework, including the Elastic Net sparse training and the WCE-mRMR channel selection strategy. Section 4 introduces the experimental design, detailing the datasets and evaluation metrics. Section 5 presents a comprehensive discussion of the experimental results, including category-wise performance, channel selection behavior, and ablation studies. Finally, Section 6 concludes the paper.

## 2. Related Work

Deep Convolutional Neural Networks (DCNNs) are the backbone of autonomous driving perception [1], but their high computational complexity limits deployment on resource-constrained Onboard Computing Units (OCUs) [9]. Various model compression techniques (quantization, low-rank factorization, knowledge distillation, network pruning) have been explored to address this [10]. Among these, structured pruning is most practical for autonomous driving: it removes entire structural units instead of individual weights, enabling direct acceleration on general-purpose hardware without specialized support—an advantage over unstructured pruning, which only offers theoretical compression [11].

### 2.1. Structural Pruning

Structured pruning differs from unstructured pruning by removing entire units, preserving network topology for seamless hardware deployment [11]. Its development addresses the flaw of equating weight magnitude with feature importance, which causes suboptimal compression and accuracy loss. This evolution has two distinct phases: magnitude-based heuristics and geometry-based redundancy mining.

Early structured pruning relied on magnitude heuristics, assuming smaller weights are more redundant [11]. Representative works include Network Slimming [13], which uses L1 regularization on BN scaling factors for sparse induction, and Soft Filter Pruning [14], which masks low-magnitude filters during training to avoid irreversible information loss. However, a critical limitation of standard magnitude pruning is the difficulty in determining optimal layer-wise sparsity. Addressing this, Lee et al. proposed LAMP (Layer-Adaptive Magnitude-based Pruning) [15], which utilizes a global importance score to automatically determine layer-wise sparsity by normalizing the squared weight magnitude against the sum of surviving weights. Despite its effectiveness in ensuring a uniform distribution of non-zero connections, LAMP fundamentally relies on physical magnitude, which inherently disadvantages the useless but informative filters associated with rare classes in long-tailed datasets.

To overcome magnitude-based limitations, research shifted to geometry-based pruning, which quantifies redundancy via feature/filter geometric properties and prunes highly replaceable filters. FPGM [16] prunes filters near the geometric median, while HRank [17] prunes filters generating low-rank, information-poor feature maps. Subsequent works extend this paradigm with distillation, reinforcement learning, and module-wise optimization [18,19,20,21]. Domain-specific applications include KSG-YOLO [22] for building object detection and Xu et al. [23] for LiDAR 3D detection. While outperforming magnitude-based methods, geometry-based pruning focuses on structural redundancy rather than semantic utility [16,18] and ignores long-tailed imbalance, leading to over-pruning of low-representation tail-class filters.

### 2.2. Information-Theoretic and Entropy-Based Feature Importance Criteria

To evaluate semantic utility, researchers use information theory, based on the Information Bottleneck principle [24]—maximizing feature-label mutual information (MI) while minimizing feature redundancy. However, traditional MI estimation in high-dimensional spaces is computationally expensive and noise-sensitive.

Recent research has introduced specialized entropy metrics to guide pruning [25]. Beyond static selection, EGSSO (Entropy-Guided Search Space Optimization) utilizes layer-wise weight entropy to refine the subnetwork search space [26], avoiding suboptimal decisions inherent in uniform pruning. Lu, Y. et al. [27] proposed an entropy-induced pruning framework, where channel importance is measured via the Shannon entropy of activation maps. Channels with low entropy are progressively pruned, followed by finetuning to recover accuracy. B. Muşat and R. Andonie proposed spatial aura entropy [28], which evaluates the heterogeneity of neural activations over a spatial neighborhood to mitigate the noise sensitivity of traditional estimators. Similarly, the FSIM-E [29] method combines feature similarity with 2D entropy to remove filters that generate low-entropy outputs or possess high low-level similarity.

A key theoretical advancement is the introduction of Copula Entropy (CE), which Ma and Sun demonstrated to be equivalent to the negative of mutual information [30]. Unlike traditional estimators that require marginal density estimation, CE separates the dependence structure from marginals, enabling efficient estimation via rank statistics [31,32]. It has been successfully applied to feature selection in complex systems, such as power grid stability prediction, by constructing image-like data to identify high-dimensional redundancy. This theoretical property makes CE uniquely positioned to capture the non-linear dependencies in deep neural networks without assuming Gaussian distributions, fitting the requirements of complex visual tasks [33,34].

### 2.3. Optimization for Long-Tailed Object Detection

Pruning techniques are critical for deploying object detection models, such as the YOLO series, on edge devices. For instance, YOLOv8n-FAWL [35] integrates LAMP with lightweight modules to enhance small object detection. However, real-world autonomous driving data typically follows a long-tailed distribution [36,37]. Standard pruning can disproportionately degrade the performance of minority classes due to their lower activation magnitudes [38]. While specific pruning methods for long-tailed data are scarce, general optimization strategies exist. MMT (Mixed Mutual Transfer) [39] facilitates knowledge transfer between head and tail classes by blending samples to reverse the long-tailed distribution.

**Figure 1:**
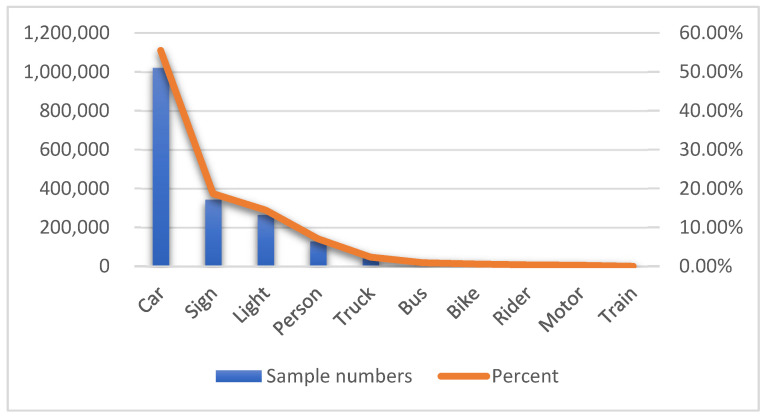
Long-tailed category distribution in the BDD100K dataset.

To contextualize the challenges of object detection in autonomous driving, we analyze the category distribution of the BDD100K dataset [12], as detailed in Table 1 and visualized in Figure 1. The dataset exhibits a pronounced long-tailed distribution, with the car class accounting for over 55% of total samples (1,021,857), while tail classes such as train represent a mere 0.0097% (179 samples). This extreme imbalance poses significant challenges for deep neural networks, as models tend to prioritize head classes at the expense of tail class accuracy. Our proposed WCE-mRMR channel scoring method is designed to address this issue by capturing non-linear dependencies between channels and task objectives, even for underrepresented classes, ensuring that critical features for both head and tail categories are retained during pruning. This is particularly evident in the channel importance scores, where channels contributing to tail class detection are weighted more heavily, leading to improved performance on low-sample categories after model compression.

Despite these notable advances, a critical research gap still persists in the existing literature: mainstream pruning methods either center on mining structural redundancy yet fail to address the inherent class imbalance in real-world autonomous driving datasets, or adopt general entropy-based evaluation metrics but lack an effective mechanism to reweight the probability space for rare-class events. To fill this gap, our work integrates two key components: Elastic Net regularization, which serves to induce robust structural sparsity and preserve the network’s structural integrity, and a novel Weighted Copula Entropy metric, which enables sample-balanced semantic feature selection tailored for long-tailed traffic scenarios.

## 3. Methods

This section details the mathematical formulation and algorithmic pipeline of the proposed channel pruning approach. First, an overview of the compression framework is provided. Subsequently, we introduce the mechanism of inducing structural sparsity via Elastic Net regularization. We then present the rigorous derivation of the class-balanced WCE, which serves to decouple feature relevance from sample abundance. Finally, we describe the integration of WCE into the mRMR search strategy to finalize the optimal pruned architecture.

### 3.1. Overview of the Pruning Framework

To address the channel redundancy inherent in Convolutional Neural Networks (CNNs) trained on long-tailed datasets like BDD100K, we propose a comprehensive two-stage compression framework. As illustrated in Figure 1, the core challenge is not merely reducing model size but identifying a subset of feature channels that maximizes the representation of minority classes while maintaining overall detection performance. The process begins with a sparse training phase, designed to induce structural sparsity in the network weights, followed by an iterative channel selection and pruning phase. Unlike traditional methods that equate parameter magnitude with importance, our approach evaluates the semantic utility of features using information theory, specifically integrating a novel Weighted Copula Entropy with the Maximum Relevance Minimum Redundancy criterion. This ensures that the preservation of discriminators is driven by their intrinsic information content regarding target classes rather than their occurrence frequency.

**Figure 2:**
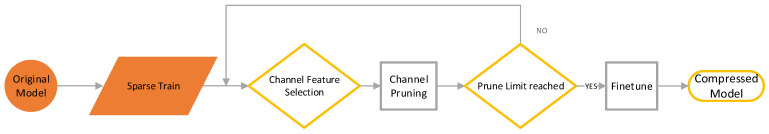
Flowchart of the proposed iterative channel pruning framework.

As illustrated in Figure 2, the proposed compression framework begins with the original model. The process initiates with a sparse train phase to induce sparsity in the network weights. Subsequently, the algorithm enters an iterative loop consisting of channel feature selection and channel pruning. In each iteration, channel importance is evaluated, and the least significant channels are removed. This cycle continues until the prune limit condition is satisfied. Finally, a finetune stage is employed to recover any accuracy loss incurred during pruning, resulting in the final compressed model.

In this study, we address the channel redundancy problem in Convolutional Neural Networks (CNNs) trained on long-tailed datasets, specifically BDD100K. The core challenge lies in identifying a subset of feature channels that maximizes the representation of minority classes while maintaining overall detection performance. To achieve this, we propose a two-stage feature selection framework.

### 3.2. Sparse Training via Elastic Net Regularization

First, to induce a sparse structure during the training phase, we modify the objective function to include an Elastic Net penalty on the vector of scaling factors on batch normalization for each layer. The regularization term is defined as Equations (1) and (2):

(1) $$L_{sparsity}=L_{org}+R(\gamma )$$

(2) $$R(\gamma )=\lambda \sum_{l=1}^{L}\left(\rho ||\gamma_{l}|{|}_{1}+(1-\rho )\frac{1}{2}||\gamma_{l}|{|}_{2}^{2}\right)$$

where λ determines the regularization strength and ρ∈[0,1] controls the mix between $L_{1}$ and $L_{2}$ penalties. This regularization drives unimportant channel scaling factors towards zero, effectively exposing the redundant structures within the network.

Second, for the selection of the final surviving channels, we depart from magnitude-based selection. Instead, we propose a feature selection algorithm that integrates mRMR with WCE. This approach ensures that the selected feature channels maintain discriminative power for minority classes despite the prevalence of majority classes. The following subsections detail the theoretical derivation of WCE and the optimization of the selection criterion.

### 3.3. Derivation of Class-Balanced Weighted Copula Entropy

#### 3.3.1. Preliminaries: Copula Entropy

Copula Entropy is a mathematically rigorous measure of statistical independence. According to Sklar’s theorem and the theory proposed by Ma and Sun [30], the Mutual Information between random variables X and Y is equivalent to the negative entropy of their corresponding copula density.

Let X be a random variable with marginal Cumulative Distribution Function (CDF) $F_{X}(x)$, and Y be a random variable with marginal CDF $F_{Y}(y)$. The copula entropy $H_{c}(X,Y)$ is defined as:

(3) $$H_{c}(X,Y)=-\int_{0}^{1}\int_{0}^{1}c(u,v)\log c(u,v)dudv$$

where $u=F_{X}(x)$, $v=F_{Y}(y)$, and c(u,v) is the copula probability density function. The relationship between MI and CE is given by:

(4) $$I(X;Y)=-H_{c}(X,Y)$$

CE has significant advantages over traditional correlation coefficients as it captures non-linear, high-order dependencies and makes no assumptions about the underlying distribution.

In the context of the BDD100K dataset, the class distribution is highly imbalanced. Standard estimation of MI via CE relies on the Empirical Cumulative Distribution Function (ECDF). However, the standard ECDF treats every sample equally, causing the estimated dependence to be dominated by head classes. To rectify this, we introduce a sample reweighting mechanism into the copula framework.

#### 3.3.2. Sample Weighting Strategy

Let $D={\left\{(x_{i},y_{i})\right\}}_{i=1}^{N}$ denote the dataset derived from the Regions of Interest (ROIs) of the feature maps, where N is the total number of samples. Let M be the number of classes. For a sample i belonging to a class m, we define the balancing weight $w_{i}$ as the inverse class frequency:

(5) $$w_{i}=\frac{1}{N_{y_{i}}}\times \frac{N}{M}$$

where $N_{y_{i}}$ is the count of samples in class $y_{i}$. The weights are normalized such that $\sum_{i=1}^{N}w_{i}=1$.

#### 3.3.3. Weighted Empirical Cumulative Distribution Function

Crucially, we propose a Weighted ECDF to replace the standard ECDF in the probability integral transform. Consequently, the transformed variables are no longer based on uniform ranks but on the cumulative weight of the samples. This mapping projects the original long-tailed distribution into a balanced Copula domain, allowing us to estimate a WCE via k-Nearest Neighbor estimators. For the numerical estimation of WCE, we adopt the widely used Kraskov-Stögbauer-Grassberger (KSG) k-nearest neighbor entropy estimator [40], which is the standard and robust approach for copula entropy-based mutual information calculation [30]. The neighborhood size k is set to 3, following the widely adopted configuration in existing copula entropy-based feature selection works, which balances estimation accuracy and computational efficiency for high-dimensional convolutional feature data.

The weighted ECDF, denoted as ${\hat{F}}_{w}(x)$. For a channel feature, the weighted ECDF at value x is defined as:

(6) $${\hat{F}}_{w}(x)=\sum_{i=1}^{N}w_{i}\cdot I(x_{i}\leq x)$$

where I(⋅) is the indicator function.

Consequently, the transformed variable ${\hat{F}}_{w}(x_{\left(i\right)})$ for the i-th sample is no longer based on the uniform rank rank(i)/N, but rather on the cumulative weight of the samples:

(7) $${\hat{F}}_{w}(x_{\left(i\right)})=\sum_{k\in \{j|x_{j}\leq x_{i}\}}w_{k}$$

#### 3.3.4. Weighted Copula Entropy Definition

By applying the weighted ECDF to both the feature variable X and the target variable Y, we map the original long-tailed distribution to a balanced Copula domain. The Weighted Copula Entropy is then estimated as:

(8) $$I_{w}(X;Y)=-H_{weighted\_copula}({\hat{F}}_{w}(X),{\hat{F}}_{w}(Y))$$

Numerically, this is implemented by applying the K-Nearest Neighbor entropy estimator on the weighted-rank transformed data. This ensures that the mutual information $I_{w}$ reflects the dependency across all classes equally, preventing the car class from masking the signal of the bicycle class.

### 3.4. Weighted mRMR for Channel Selection

To finalize the pruning structure, we embed this weighted metric into a greedy forward search strategy based on the mRMR principle. The objective is to select a subset of channels that maximizes dependency with the target labels while minimizing the mutual information shared among the selected channels themselves. By calculating both relevance and redundancy using our WCE, we ensure that a channel is not discarded simply because it shares information about the majority classes. It is only penalized if it is redundant regarding the balanced class set. The algorithm iteratively selects features that offer the highest marginal gain in information density, resulting in a compressed model that retains high discriminative power for both head and tail classes.

The objective is to select a subset of channels S from the complete feature map $F=\{f_{1},f_{2},\ldots ,f_{M}\}$ that maximizes the dependency with the target Y while minimizing internal redundancy. We adapt the mRMR criterion using our derived $I_{w}$.

#### 3.4.1. Max-Relevance

We seek channels that have the highest mutual information with the class labels under the balanced distribution. The relevance D of a subset S is defined as:

(9) $$D(S,Y)=\frac{1}{\left|S\right|}\sum_{f_{i}\in S}I_{w}(f_{i};Y)$$

#### 3.4.2. Min-Redundancy

We aim to minimize the mutual information between selected channels. Crucially, we use $I_{w}$ for redundancy as well, ensuring that channels are not considered redundant simply because they share information about the majority classes; they must share information regarding the balanced class set. The redundancy R is defined as:

(10) $$R(S)=\frac{1}{|S{|}^{2}}\sum_{f_{i},f_{j}\in S}I_{w}(f_{i};f_{j})$$

#### 3.4.3. Optimization Criterion

We employ a greedy forward search strategy. Given an already selected set $S_{m-1}$ with m−1 features, the m feature $f_{j}$ is selected from the remaining set $F\setminus S_{m-1}$ by maximizing the following objective:

(11) $$\max_{f_{j}\in F\setminus S_{m-1}}\left(I_{w}(f_{j};Y)-\frac{1}{\left|S_{m-1}\right|}\sum_{f_{k}\in S_{m-1}}I_{w}(f_{j};f_{k})\right)$$

By iteratively solving this equation, we obtain a subset of channels that are highly discriminative for the BDD100K object detection task across all categories, effectively mitigating the bias introduced by the long-tailed distribution.

To materialize the weighted mRMR optimization criterion defined in Equation (11) for channel selection, we formalize the entire process into a greedy forward search algorithm, denoted as channel selection via weighted copula entropy. This algorithm integrates the sample weighting strategy, weighted ECDF calculation and WCE-based mutual information estimation into the mRMR framework, iteratively selecting the channel with the maximum marginal information gain until the preset number of reserved channels K is reached. All relevance and redundancy calculations in the algorithm are based on WCE, which ensures that the selection result is immune to the long-tailed bias of traffic data and retains the discriminative features for tail classes.

The optimal channel subset *S* generated by Algorithm 1 provides an information-theoretic channel importance ranking, which is further fused with the structural sparsity score from Elastic Net regularization via element-wise summation for the final pruning.
**Algorithm 1** Channel Selection via Weighted Copula Entropy**Input:** Feature map tensor $X\in {\mathbb{R}}^{N\times C\times H\times W}$ Target labels $Y\in {\mathbb{R}}^{N}$ Number of channels to select *K* **Output:** Selected channel indices subset *S*1. Flatten spatial dimensions of *X* to obtain feature matrix $F\in {\mathbb{R}}^{N\times C}$2. Compute sample weights *W* by Equation (5);3. **for** each channel j∈{1,…,C}
**do**4. Compute Weighted ECDF ${\hat{F}}_{j}(x)$ using weights *W* by Equation (7);5. Estimate Mutual Information $I(F_{j};Y)$ by Equation (8);6. **end for**7. mRMR Greedy Search selected set S←∅, candidate set Ω←{1,…,C}8. **while**
|S|<K
**do**9. $score_{best}\leftarrow -\infty$ & $j_{best}\leftarrow \mathrm{null}$10. **for** each candidate channel j∈Ω
**do**11. Calculate relevance *D* by Equation (9);12. Calculate redundancy *R* by Equation (10);13. Objective Function Φ(j)←D−R by Equation (11);14. **if** Φ(j) > $score_{best}$
**then**15. $score_{best}\leftarrow \Phi (j)$ & $j_{best}\leftarrow j$16. **end if**17. **end for**18. $S\leftarrow S\cup \{j_{best}\}$ & $\Omega \leftarrow \Omega \setminus \{j_{best}\}$19. **end while**20. Return *S*

**Figure 3:**
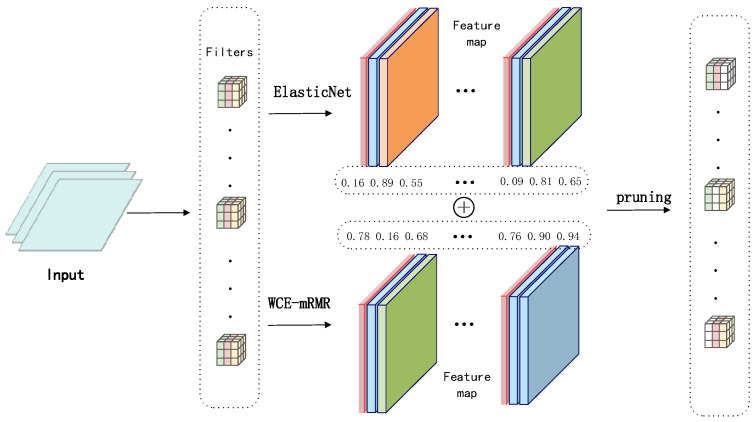
Illustration of the score fusion strategy combining ElasticNet and WCE-mRMR.

Figure 3 illustrates the channel feature selection mechanism. To enable a robust assessment of channel importance, the input feature maps are analyzed via a dual-criteria framework. Specifically, we employ two complementary methods: Elastic Net for regularization and feature grouping capabilities, and WCE-mRMR for its information-theoretic channel evaluation. To eliminate the numerical scale gap between the two complementary scores, we perform layer-wise min-max normalization to map both scores into the unified interval [0, 1] independently within each convolutional layer. This normalization ensures fair and unbiased fusion of the two metrics, avoiding dominance from the score with a wider numerical range. The final fused importance score for the *i*-th channel in the *l*-th layer is calculated as:

(12) $$S_{\mathrm{final}}(i,l)=\partial \cdot S_{\mathrm{WCE}\text{-}\mathrm{norm}}(i,l)+(1-\partial )\cdot S_{\mathrm{EN}\text{-}\mathrm{norm}}(i,l)$$

where $S_{\mathrm{EN}\text{-}\mathrm{norm}}(i,l)$ and $S_{\mathrm{WCE}\text{-}\mathrm{norm}}(i,l)$ represent the normalized Elastic Net sparsity score and normalized WCE-mRMR information score, respectively. The balance coefficient ∂ is set to 0.5 by default for equal-weight fusion, whose optimality and robustness are verified via sensitivity analysis in the ablation study. Channels with lower final fused scores are pruned preferentially in each iterative pruning step.

## 4. Experiments Settings

This section outlines the experimental setup used to validate the effectiveness of the proposed WCE pruning framework. We describe the baseline models, the hardware and software environments, and the evaluation metrics employed to assess both computational efficiency and detection accuracy. Furthermore, we detail the characteristics of the large-scale BDD100K driving dataset utilized in our evaluations.

### 4.1. Experimental Details

To validate our Weighted Copula Entropy channel pruning framework, we conduct standardized comparisons against L1 [13], HRank [17], FPGM [15], and LAMP [16] on YOLOv8l/YOLOv5l using the BDD100K dataset [12]. These baselines represent established structured pruning approaches, while YOLO variants ensure real-world accuracy-efficiency balance. All experiments use identical hyperparameters for fair evaluation.

We implement our method using Python 3.9.18, Pytorch 2.0.1, CUDA 12.1, and cuDNN 8.9.2. All experiments follow the same experimental settings and evaluation metrics, and all experiments are run on a computer equipped with an Intel Xeon Silver 4116 CPU processor, 128 GB of memory, and an NVIDIA RTX8000.

### 4.2. Evaluation Metrics

In order to validate the effectiveness of the model proposed in this work, the following metrics were introduced to evaluate the model.

Mean Average Precision (mAP): The mAP refers to averaging the AP over all categories. The AP is called the mean accuracy, which is an average of the accuracies at different recall points. mAP@0.5 represents the mean Average Precision calculated at an Intersection over Union (IoU) threshold of 0.5

Floating Point Operations (FLOPs): FLOPs represent the total number of floating-point arithmetic operations executed by a neural network during the inference process. FLOPs directly reflect the computational overhead of the model, making it crucial for assessing the efficiency of model compression and optimization strategies.

Pruning Ratio (pr): Pruning Ratio is the fraction of channels removed, enabling quantitative evaluation of compression-accuracy trade-offs. It can be expressed as Pruning Ratio = (Number of Removed Parameters/Total Parameters in Original Model) × 100%.

Parameters (Params): Params denote the total number of trainable parameters in a neural network, such as weights and biases. As a key metric for assessing model complexity, Params reflects the model’s storage and memory footprint and is essential for evaluating the effectiveness of parameter compression in pruning algorithms.

### 4.3. Dataset

The BDD100K dataset is designed to support the research and development of autonomous vehicles, especially in computer vision tasks such as object detection, semantic segmentation, lane detection, and more. The BDD100K dataset comprises over 100,000 driving videos, totaling more than 1000 h of driving footage. The labels in the dataset encompass 10 categories: Bus, Traffic Light, Traffic Sign, Person, Bike, Truck, Motor, Car, Train, and Rider. The dataset covers a variety of weather conditions, lighting conditions, urban/rural environments, and different times of day (daytime, nighttime).

## 5. Experimental Results and Discussion

Building upon the experimental design established in Section 4, this section presents a comprehensive evaluation of the proposed method. We first provide an overall performance comparison against the pruning baselines, followed by a detailed category-wise analysis to demonstrate the framework’s robustness in long-tailed scenarios. Additionally, we analyze the distinct channel selection behavior of our metric and provide qualitative visual evidence through feature maps and detection bounding boxes. Finally, an ablation study is presented to validate the contribution of each core component within our framework.

### 5.1. Overall Performance Comparison

**Figure 4:**
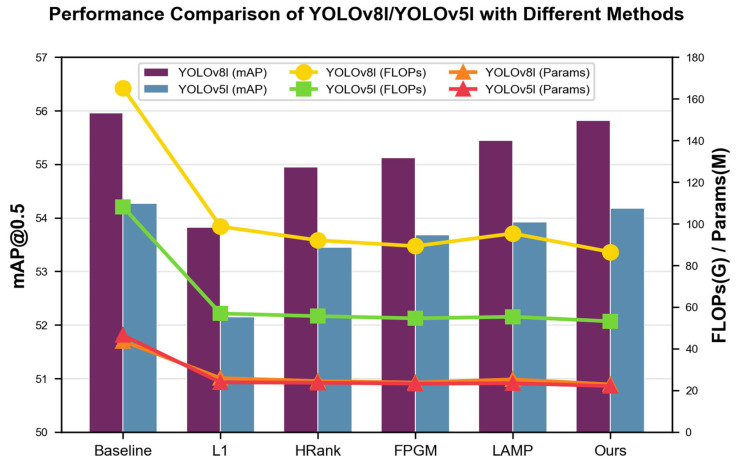
Performance of YOLOv8l and YOLOv5l under different pruning methods.

Table 2 and Figure 4 present the overall performance of different structured channel pruning methods on YOLOv8l and YOLOv5l at a 50% pruning ratio, evaluated on the BDD100K dataset. The results are reported in terms of mAP@0.5, FLOPs, and the number of parameters, which are the key metrics for assessing detection accuracy, computational complexity, and model size, respectively.

As shown in Table 2, the proposed WCE-based pruning method achieves the most favorable trade-off between accuracy and efficiency. For YOLOv8l, the baseline mAP@0.5 is 55.96%. After pruning, our method only incurs a minimal accuracy drop of 0.14% (to 55.82%), while reducing FLOPs from 165.2 G to 86.5 G and Params from 43.7 M to 22.9 M. In comparison, other methods such as L1, HRank, FPGM, and LAMP suffer from significantly larger accuracy losses, ranging from 1.01% to 2.14%, while achieving less pronounced compression. Similarly, for YOLOv5l, our method maintains a mAP@0.5 of 54.18%, only a 0.09% drop from the baseline of 54.27%, and reduces FLOPs to 53.2 G and Params to 22.1 M, outperforming all competing methods. These results demonstrate that our approach effectively preserves detection accuracy while substantially reducing the computational and memory overhead of the models.

To better visualize the trade-offs between detection accuracy and computational efficiency, Figure 4 presents a comprehensive performance comparison of YOLOv8l and YOLOv5l under different pruning methods at a 50% pruning rate. The figure employs a dual-axis design: the left axis represents mAP@0.5 via bar charts, while the right axis displays FLOPs and the number of parameters via line charts.

### 5.2. Category-Wise Performance Analysis

To further investigate the robustness of the proposed method under the long-tailed data distribution in autonomous driving scenarios, we provide a category-wise mAP comparison in Table 3. This analysis focuses on the performance of each method across different object categories, including both frequent, large-sample classes (e.g., Car, Traffic Sign) and infrequent, small-sample classes (e.g., Train, Rider).

From Table 3, it is evident that our method exhibits superior performance, especially on small-sample and hard-to-detect categories. For instance, on YOLOv8l, the mAP of the Train class under our method is 5.12%, which is significantly higher than the baseline of 2.76% and all other pruning methods. Similarly, on YOLOv5l, our method achieves a mAP of 3.84% for the Train class, while the baseline is 0%, and other methods either remain at 0% or show only marginal improvements. For the more frequent classes, our method maintains competitive mAP values that are slightly lower than the baseline but consistently higher than those of the other pruning methods. This indicates that our approach not only mitigates the performance degradation on small-sample classes, which is a common issue in traditional pruning methods, but also preserves the detection accuracy on large-sample classes effectively.

In summary, the experimental results demonstrate that the proposed WCE-based structured channel pruning method achieves State-of-the-Art performance in terms of both overall accuracy-efficiency trade-off and category-wise robustness. It effectively addresses the challenges of model compression in autonomous driving scenarios, where both efficiency and detection performance on long-tailed data are critical.

### 5.3. Channel Selection Behavior Analysis

**Figure 5:**
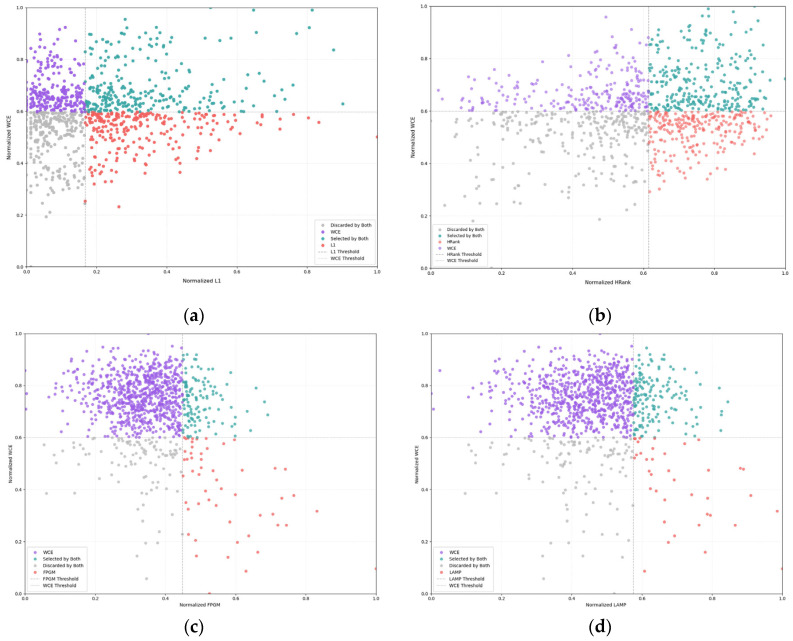
Scatter plots of normalized WCE vs. normalized scores of (**a**) L1, (**b**) HRank, (**c**) FPGM, and (**d**) LAMP, illustrating channel selection behavior and overlap between our method and baseline pruning strategies.

To further analyze the channel selection behavior of the proposed WCE method and its differences from traditional pruning strategies, we present scatter plots of normalized WCE scores against the normalized scores of L1, HRank, FPGM, and LAMP in Figure 5. Specifically, this analysis is conducted on a single convolutional layer with 1024 channels from the YOLOv5l model at a 50% pruning rate, ensuring the analysis focuses on a representative and high-dimensional layer typical of modern object detection architectures. Each subplot corresponds to one baseline method, with the x-axis representing the normalized score of the baseline method and the y-axis representing the normalized WCE score. The dashed vertical and horizontal lines denote the 50% pruning rate thresholds for the baseline method and WCE, respectively, dividing the space into four quadrants that represent different channel selection outcomes:

- Teal points: Channels selected by both the baseline method and WCE.
- Purple points: Channels selected only by WCE and discarded by the baseline method.
- Red points: Channels discarded only by WCE and selected by the baseline method.
- Gray points: Channels discarded by both methods.

From the scatter plots of the 1024-channel layer in YOLOv5l, several key observations emerge. First, WCE retains numerous channels discarded by baselines in low-scoring regions—specifically for long-tailed categories, where magnitude-based methods fail due to heuristic scoring biases. This directly enables superior minority-class detection, as these channels encode discriminative features for rare objects. Second, substantial teal-point overlap in high-scoring regions confirms WCE’s alignment with baselines on head-class channels, ensuring no performance degradation on frequent categories. Crucially, the concentration of purple points in low-baseline-score zones demonstrates WCE’s information-theoretic advantage: it prioritizes channels with high information value, thereby mitigating accuracy degradation from long-tailed data distributions. By simultaneously preserving head-class robustness and recovering minority-class fidelity, our method ensures a safety advantage for autonomous driving systems operating under extreme class imbalance.

This visualization of the 1024-channel layer in YOLOv5l provides a clear and intuitive demonstration of the unique channel selection mechanism of WCE. Unlike traditional methods that rely on simple heuristic scores to rank all 1024 channels, WCE leverages the copula entropy of feature maps across all categories, enabling it to identify and preserve channels that are essential for maintaining detection performance on both frequent and infrequent object classes.

While the proposed WCE framework achieves a superior trade-off between detection accuracy and inference efficiency, it is necessary to discuss its computational overhead during the training phase. The *k*-NN-based empirical copula estimation and the pairwise mRMR search are inherently more computationally intensive than simple magnitude-based ranking. To optimize this, we utilized high-degree parallel computing on a workstation equipped with an Intel Xeon Silver 4116 CPU processor and an NVIDIA RTX8000 GPU. Under this hardware configuration, each comprehensive WCE-mRMR channel selection step requires approximately 28 min for YOLOv5l and 32 min for YOLOv8l. When evaluated within the context of the end-to-end training pipeline on the large-scale BDD100K dataset, this search process introduces only a marginal overhead, increasing the overall training time by less than 5%. Since this structural search is strictly a one-time offline process prior to deployment, the slight increase in pre-deployment computational cost is highly justified by the critical gains in minority-class detection performance.

### 5.4. Analysis of Feature Representation and Detection Performance

**Figure 6:**
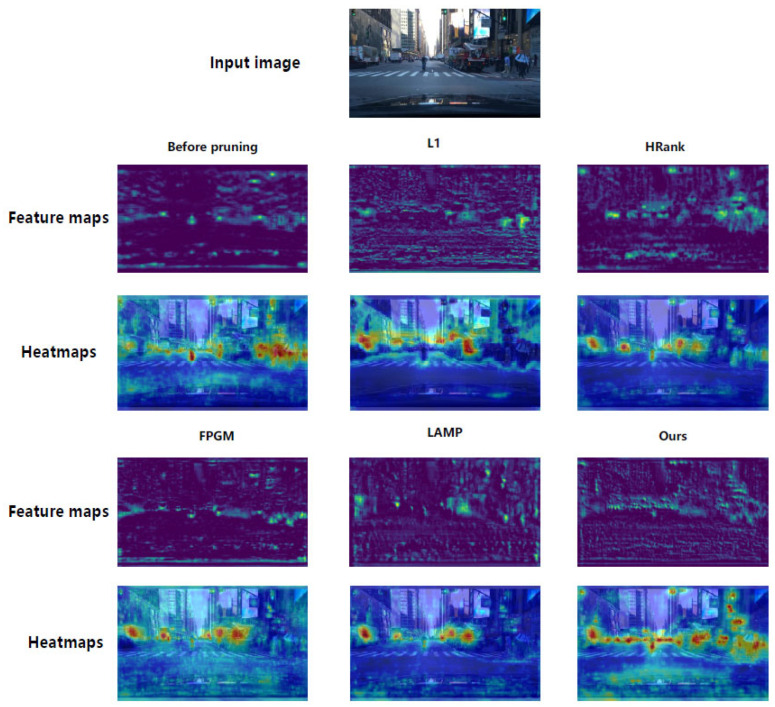
Comparison of feature maps and heatmaps across different pruning methods.

The aforementioned quantitative analysis and channel selection behavior exploration have verified the superiority of the proposed WCE method in accuracy-efficiency trade-off and long-tailed category adaptation from a numerical and structural perspective. To further reveal the intrinsic reason for such performance advantages, we conduct a qualitative analysis of the feature representation capability of different pruning methods via feature maps and heatmaps, and validate the practical detection reliability in real autonomous driving scenarios with extreme long-tailed distribution (e.g., nighttime scenes containing tail classes such as Train and Rider). The experimental results are visualized in Figure 6 and the qualitative detection comparison of typical long-tailed scenes, respectively.

As shown in Figure 6, the input image presents a complex urban driving scene featuring a rider that is crucial for autonomous driving safety but often neglected by conventional pruning methods. Feature maps from other approaches are noisy and lack clear activation patterns, especially for the rider. Their activation heatmaps further highlight limitations: the responses to the rider are weak, scattered, or entirely absent. This issue arises because standard pruning methods conflate low feature magnitude with unimportance, inadvertently pruning channels critical for minority-class detection. In contrast, our method produces cleaner feature maps with structured activation patterns. The corresponding heatmaps exhibit strong, focused activations precisely aligned with key traffic participants.

**Figure 7:**
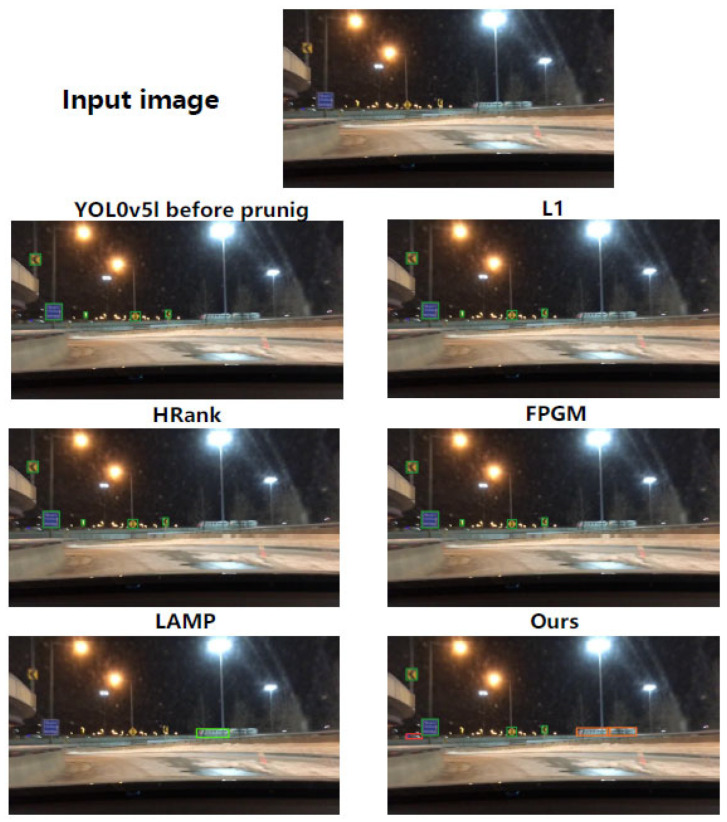
Detection results of YOLOv5l on a nighttime scene with the tail class Train.

To further demonstrate the practical effectiveness of our pruning method in maintaining detection accuracy for critical minority classes, we provide a qualitative comparison via Figure 7. The input image shows a nighttime autonomous driving scene featuring a train, a class that is extremely underrepresented in the BDD100K dataset, which poses significant challenges to both feature extraction and pruning strategies.

Notably, the original YOLOv5l model fails to detect the train, and models pruned using methods such as L1 norm, HRank, and FPGM also fail to identify it, as channel pruning discards discriminative features crucial for recognizing rare targets. Although LAMP detects the train, it sacrifices detection of other critical traffic objects, demonstrating an unacceptable trade-off between minority-class detection and overall reliability.

In contrast, our method not only accurately detects the train but also robustly identifies all other visible traffic objects. This is attributed to the class-balanced weighting mechanism in our channel scoring framework. WCE-mRMR ensures that channels encoding discriminative information for minority classes like trains are retained during the pruning process, without sacrificing the features necessary for reliably detecting other critical targets. As a result, our pruned model reduces computational complexity while preserving the comprehensive detection reliability required for safe autonomous driving.

**Figure 8:**
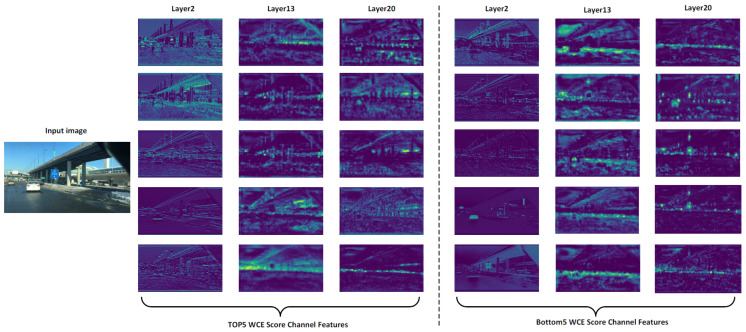
Feature utility diagnosis of the unpruned model based on Weighted Copula Entropy.

To further understand how the unpruned network encodes information, we utilized the proposed WCE not merely as a pruning criterion, but as an independent feature-utility diagnostic tool. Figure 8 visualizes the feature maps from channels with the Top-5 and Bottom-5 WCE scores across shallow, intermediate, and deep layers of the unpruned YOLOv5l network.

The input image presents a complex, heavily imbalanced autonomous driving scene; it contains abundant majority-class objects (e.g., numerous cars and traffic signs), while a critical minority-class train is located in the middle-upper right region. The visualization reveals a stark contrast in semantic encoding. In the shallow layer, the Top-5 WCE channels explicitly capture crucial low-level structural boundaries of the overpass and vehicles.

As the features undergo high-level abstraction in deeper layers, the diagnostic capability of WCE becomes profoundly evident. Despite the visual dominance of the cars and traffic signs in the foreground, the Top-5 WCE channels effectively suppress these frequent patterns and exhibit highly concentrated, semantically meaningful activations precisely anchored on the train in the middle-upper right area.

In contrast, the Bottom-5 WCE channels in deep layers demonstrate profound feature collapse. The resulting activation maps consist largely of diffuse background noise or zero-activation regions, offering no semantic utility. By serving as a diagnostic lens, this analysis reveals a critical intrinsic property of the unpruned YOLOv5l model. It naturally reserves specific, high-utility channels to encode vulnerable tail classes despite overwhelming head-class interference. The proposed WCE metric successfully decodes this mechanism, isolating these information-dense features with high precision.

### 5.5. Ablation Study

To quantitatively verify the effectiveness and necessity of each core component in the proposed Weighted Copula Entropy pruning framework, we conduct ablation experiments on YOLOv5l and YOLOv8l with a 50% pruning rate on the BDD100K dataset (Table 4). The framework’s key modules include Elastic Net regularization, class-balanced (CB) weighting, long-tailed data adaptation, WCE, and weighted mRMR. We construct four variant models by ablating different components and compare their performance with the complete framework, using overall mAP@0.5, FLOPs, and Params as evaluation metrics.

All experimental settings are kept identical to ensure fair comparison, with four ablation variants designed as follows:

1. Elastic Net Only: Only apply Elastic Net regularization on BN scaling factors for sparse training, and prune channels by weight magnitude.
2. WCE *w*/*o* CB: Integrate Elastic Net and raw Copula Entropy for channel scoring, with the class-balanced inverse frequency weighting ablated.
3. WCE + CB *w*/*o* mRMR: Retain Elastic Net, CB weighting, and WCE for feature relevance evaluation, with the weighted mRMR module ablated.
4. Complete WCE Framework: The full method fusing Elastic Net, CB-weighted WCE, and weighted mRMR for channel selection and pruning.

Key conclusions on component contributions are drawn as follows:

1. Elastic Net is the foundation of structural sparsity: This variant achieves basic model compression but yields the lowest overall, as magnitude-based pruning neglects discriminative features in long-tailed traffic data, verifying the insufficiency of pure sparse regularization without semantic feature evaluation.
2. WCE provides a more rational feature importance metric: Compared to Elastic Net Only, the WCE *w*/*o* CB variant achieves a clear improvement in overall mAP@0.5 (YOLOv5l: +0.65%, YOLOv8l: +0.54%) while slightly reducing FLOPs and Params. This demonstrates that by evaluating channel importance based on copula entropy, rather than just weight magnitude, WCE can better identify and retain channels that encode meaningful semantic information, leading to more accurate and efficient pruning decisions.
3. CB weighting is critical for long-tailed adaptation: Compared with WCE *w*/*o* CB, WCE + CB *w*/*o* mRMR achieves a further significant improvement in mAP@0.5 (YOLOv5l: +0.46%, YOLOv8l: +0.52%) and more pronounced reductions in FLOPs and Params. The inverse class frequency weighting embedded in the empirical copula estimation effectively decouples feature relevance from sample abundance, enabling WCE to capture the semantic information of minority class features that are otherwise masked by majority classes in the raw CE calculation. This validates our core design for adapting to the long-tailed distribution of traffic data.
4. Weighted mRMR eliminates semantic redundancy: The complete framework further improves overall mAP@0.5 (YOLOv5l: +0.21%, YOLOv8l: +0.23%) while achieving the lowest FLOPs and Params across all variants. Weighted mRMR not only selects channels with high WCE relevance but also minimizes semantic redundancy between them, forming a more compact and diverse feature set. This optimization enhances both overall detection accuracy and the discriminative power of the pruned model, while delivering more efficient compression.
5. Synergistic fusion of components achieves optimal performance: The full WCE framework integrates the advantages of all modules, realizing the highest overall mAP@0.5 with the optimal compression efficiency (lowest FLOPs and Params) for both YOLOv5l and YOLOv8l. This fully verifies the rationality of the modular design, where each component is indispensable, and their fusion addresses the core limitations of traditional pruning methods in long-tailed autonomous driving scenarios.

### 5.6. Sensitivity Analysis of Fusion Weight

To verify the rationality of the default fusion weight α = 0.5 and the robustness of the score fusion mechanism, we conduct a sensitivity analysis on YOLOv5l and YOLOv8l with a 50% pruning rate. We adjust the balance coefficient α from 0 to 1 with a step size of 0.1 and record the overall mAP@0.5 under different settings.

**Figure 9:**
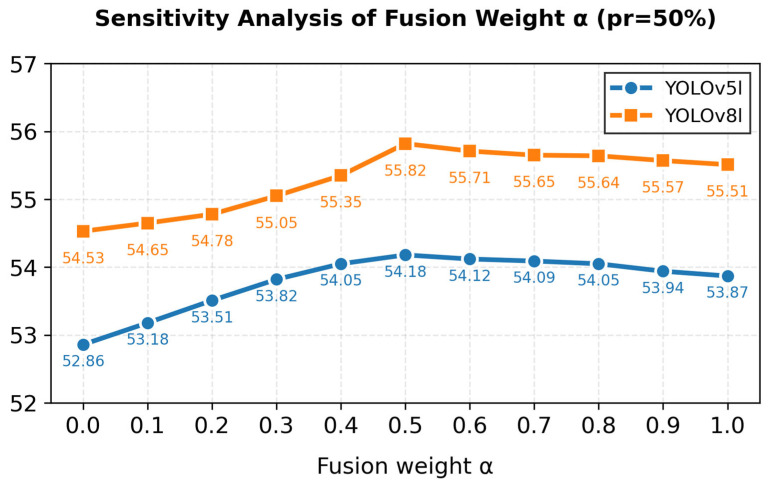
Sensitivity analysis of the fusion weight α (pr = 50%).

As illustrated in Figure 9, the mAP@0.5 of both models exhibits a consistent unimodal trend with the increase in α. It rises gradually as α increases from 0.0 to 0.5, and decreases slowly when α exceeds 0.5. Specifically, YOLOv5l achieves the optimal mAP@0.5 of 54.18% at α = 0.5, while YOLOv8l reaches its peak performance (55.82% mAP@0.5) at the same fusion weight. This result verifies the rationality of the default equal-weight fusion strategy, where the structural sparsity guided by Elastic Net and the semantic relevance measured by WCE-mRMR are balanced effectively, avoiding the dominance of a single scoring metric.

## 6. Conclusions

Traditional structured pruning often equates parameter magnitude with semantic feature value, causing critical over-pruning of minority-class discriminative channels in autonomous driving’s long-tailed scenarios. To address this, we propose a Weighted Copula Entropy framework that decouples feature relevance from sample abundance through three synergistic components: Elastic Net regularization, class-balanced inverse frequency weighting, and weighted mRMR. This framework enables class-balanced information-theoretic feature selection that is robust to the inherent sample imbalance of the training data, while simultaneously inducing network sparsity. Evaluated on YOLOv5l/YOLOv8l with BDD100K, our method achieves negligible mAP degradation with ~50% FLOPs reduction, while significantly boosting extreme minority-class detection. Ablations confirm the necessity of all core modules. Limitations include monomodal application and fixed pruning ratios. Future work will extend to multi-modal perception, dynamic pruning rates, and integrated compression via quantization/knowledge distillation. This framework delivers a practical solution for lightweight autonomous perception and establishes a novel information-theoretic paradigm for long-tailed visual recognition.

## Figures and Tables

**Table 1:** Category distribution of object detection samples in the BDD100K dataset.

Class	Sample Numbers	Percent
Car	1021,857	55.49%
Traffic Sign	343,777	18.67%
Traffic Light	265,906	14.44%
Person	129,262	7.02%
Truck	42,963	2.33%
Bus	16,505	0.90%
Bike	10,229	0.56%
Rider	6461	0.35%
Motor	4296	0.23%
Train	179	0.01%

**Table 2:** Overall performance comparison of different pruning methods on YOLOv8l and YOLOv5l.

Model	Method	mAP@0.5 (%)	FLOPs (G) (pr = 50%)	Params (M)
YOLOv8l	Baseline	55.96 ± 0.21	165.22	43.71
	L1	53.82 ± 0.37	98.73	25.92
	HRank	54.95 ± 0.30	92.09	24.53
	FPGM	55.12 ± 0.32	89.28	23.76
	LAMP	55.45 ± 0.27	95.36	25.33
	Ours	55.82 ± 0.18	**8** **6** **.5**	**2** **2** **.** **87**
YOLOv5l	Baseline	54.27 ± 0.24	108.12	46.52
	L1	52.15 ± 0.39	56.94	23.89
	HRank	53.45 ± 0.35	55.67	23.68
	FPGM	53.68 ± 0.29	54.63	23.27
	LAMP	53.92 ± 0.28	55.36	23.45
	Ours	54.18 ± 0.19	**53.2** **1**	**22.1** **4**

Note: The mAP results are reported as mean ± standard deviation, calculated over five independent runs with different random seeds. Bold text indicates the best (optimal) performance among all compared methods in the corresponding column.

**Table 3:** Category-wise comparison of different pruning methods on YOLOv8l and YOLOv5l.

Model	Classs	Baseline (mAP)	L1	HRank	FPGM	LAMP	Ours
YOLOv8l	Car	80.25 ± 0.13	78.12 ± 0.21	78.56 ± 0.16	78.83 ± 0.16	79.17 ± 0.15	79.58 ± 0.11
	Traffic Sign	70.18 ± 0.16	67.95 ± 0.23	68.42 ± 0.22	68.69 ± 0.18	69.03 ± 0.19	69.49 ± 0.13
	Traffic Light	69.36 ± 0.14	67.13 ± 0.29	67.59 ± 0.26	67.86 ± 0.24	68.20 ± 0.19	68.67 ± 0.12
	Person	66.02 ± 0.20	63.89 ± 0.34	64.35 ± 0.30	64.62 ± 0.27	64.96 ± 0.26	65.33 ± 0.19
	Truck	59.87 ± 0.21	57.64 ± 0.46	58.10 ± 0.39	58.37 ± 0.32	58.71 ± 0.29	59.18 ± 0.21
	Bus	57.63 ± 0.31	55.40 ± 0.45	55.86 ± 0.41	56.13 ± 0.40	56.47 ± 0.36	**58.05 ± 0.27**
	Bike	52.89 ± 0.33	50.66 ± 0.61	51.12 ± 0.54	51.39 ± 0.43	51.73 ± 0.42	**53.22 ± 0.28**
	Rider	49.45 ± 0.45	47.22 ± 0.65	47.68 ± 0.60	47.95 ± 0.57	48.29 ± 0.46	**49.81 ± 0.35**
	Motor	48.26 ± 0.44	46.03 ± 0.79	46.49 ± 0.71	46.76 ± 0.58	47.10 ± 0.56	**48.65 ± 0.44**
	Train	2.76 ± 0.58	2.11 ± 0.82	2.34 ± 0.74	2.47 ± 0.71	2.60 ± 0.65	**5.12 ± 0.47**
YOLOv5l	Car	79.47 ± 0.14	77.15 ± 0.19	78.75 ± 0.17	79.01 ± 0.17	78.76 ± 0.14	78.85 ± 0.10
	Traffic Sign	69.29 ± 0.15	66.89 ± 0.27	68.19 ± 0.24	68.47 ± 0.21	68.93 ± 0.19	67.95 ± 0.14
	Traffic Light	68.50 ± 0.18	66.12 ± 0.28	67.62 ± 0.25	67.60 ± 0.24	66.96 ± 0.22	67.33 ± 0.13
	Person	65.22 ± 0.19	62.98 ± 0.40	64.08 ± 0.35	64.46 ± 0.28	64.52 ± 0.26	63.87 ± 0.18
	Truck	59.25 ± 0.22	56.87 ± 0.42	58.27 ± 0.37	58.85 ± 0.36	58.51 ± 0.34	58.22 ± 0.25
	Bus	56.18 ± 0.29	53.76 ± 0.54	55.06 ± 0.47	55.24 ± 0.37	55.60 ± 0.35	**56.31 ± 0.24**
	Bike	51.01 ± 0.38	48.65 ± 0.59	49.75 ± 0.55	50.13 ± 0.49	50.39 ± 0.45	**51.11 ± 0.33**
	Rider	47.72 ± 0.46	45.53 ± 0.75	46.93 ± 0.61	47.11 ± 0.58	47.04 ± 0.49	**47.77 ± 0.37**
	Motor	46.50 ± 0.53	44.55 ± 0.78	45.85 ± 0.74	45.93 ± 0.61	46.12 ± 0.60	**46.55 ± 0.45**
	Train	0.00 ± 0.00	0.00 ± 0.00	0.00 ± 0.00	0.00 ± 0.00	2.27 ± 0.68	**3.84 ± 0.46**

Bold text indicates the best (optimal) performance among all compared methods in the corresponding column.

**Table 4:** Ablation study results of the WCE framework on YOLOv5l and YOLOv8l (pr = 50%).

Model	Variant	mAP@0.5 (%)	FLOPs (G)	Params (M)
YOLOv5l	Elastic Net Only	52.86	54.12	22.51
	WCE *w*/*o* CB	53.51	53.78	22.32
	WCE + CB w/o mRMR	53.97	53.37	22.16
	Complete WCE Framework	54.18	53.23	22.07
YOLOv8l	Elastic Net Only	54.53	89.73	23.52
	WCE *w*/*o* CB	55.07	88.24	23.09
	WCE + CB *w*/*o* mRMR	55.59	87.09	22.68
	Complete WCE Framework	55.82	86.47	22.86

## Data Availability

Data are contained within the article.

## References

[1] (2021). Taxonomy and Definitions for Terms Related to Driving Automation Systems for On-Road Motor Vehicles.

[2] Valverde M., Moutinho A., Zacchi J.-V. (2025). A Survey of Deep Learning-Based 3D Object Detection Methods for Autonomous Driving Across Different Sensor Modalities. Sensors.

[3] Wang J., Sun H., Zhu C. (2023). Vision-Based Autonomous Driving: A Hierarchical Reinforcement Learning Approach. IEEE Trans. Veh. Technol..

[4] Awan M., Whangbo T.K., Shin J. (2025). Deep learning methods for autonomous driving scene understanding tasks: A review. Expert Syst. Appl..

[5] Redmon J., Divvala S., Girshick R., Farhadi A. You Only Look Once: Unified, Real-Time Object Detection. Proceedings of the IEEE Conference on Computer Vision and Pattern Recognition (CVPR).

[6] Bochkovskiy A., Wang C.Y., Liao H.Y.M. (2020). YOLOv4: Optimal Speed and Accuracy of Object Detection. arXiv.

[7] Sapkota R., Flores-Calero M., Qureshi R., Badgujar C., Nepal U., Poulose A., Zeno P., Vaddevolu U.B.P., Khan S., Shoman M. (2025). YOLO Advances to Its Genesis: A Decadal and Comprehensive Review of the You Only Look Once (YOLO) Series. Artif. Intell. Rev..

[8] Wang J., Wu Q.M.J., Zhang N., Suto K., Zhong L. (2025). Compressing Multi-Task Model for Autonomous Driving via Pruning and Knowledge Distillation. arXiv.

[9] Zhang Y., Zhang J., Du F., Kang W., Wang C., Li G. (2025). Real-Time Lightweight Vehicle Object Detection via Layer-Adaptive Model Pruning. Electronics.

[10] Dantas P.V., Sabino da Silva W., Cordeiro L.C., Carvalho C.B. (2024). A comprehensive review of model compression techniques in machine learning. Appl. Intell..

[11] Cheng H., Zhang M., Shi J.Q. (2024). A Survey on Deep Neural Network Pruning: Taxonomy, Comparison, Analysis, and Recommendations. IEEE Trans. Pattern Anal. Mach. Intell..

[12] Yu F., Chen H., Wang X., Xian W., Chen Y., Liu F., Madhavan V., Darrell T. BDD100K: A Diverse Driving Dataset for Heterogeneous Multitask Learning. Proceedings of the IEEE/CVF Conference on Computer Vision and Pattern Recognition (CVPR).

[13] Liu Z., Li J., Shen Z., Huang G., Yan S., Zhang C. Learning Efficient Convolutional Networks through Network Slimming. Proceedings of the IEEE International Conference on Computer Vision (ICCV).

[14] Xu X., Chen Q., Xie L., Su H. Batch-Normalization-based Soft Filter Pruning for Deep Convolutional Neural Networks. Proceedings of the 16th International Conference on Control, Automation, Robotics and Vision (ICARCV).

[15] Lee J., Park S., Mo S., Ahn S., Shin J. (2020). Layer-adaptive Sparsity for the Magnitude-based Pruning. arXiv.

[16] He Y., Liu P., Wang Z., Hu Z., Yang Y. Filter Pruning via Geometric Median for Deep Convolutional Neural Networks Acceleration. Proceedings of the IEEE/CVF Conference on Computer Vision and Pattern Recognition (CVPR).

[17] Lin M., Ji R., Wang Y., Zhang Y., Zhang B., Tian Y., Shao L. HRank: Filter Pruning Using High-Rank Feature Map. Proceedings of the IEEE/CVF Conference on Computer Vision and Pattern Recognition (CVPR).

[18] Lin W., Tang S., Yu C., Ye P., Chen T., Yu C. S2HPruner: Soft-to-Hard Distillation Bridges the Discretization Gap in Pruning. Proceedings of the Advances in Neural Information Processing Systems (NeurIPS).

[19] Ganjdanesh A., Gao S., Huang H. Jointly Training and Pruning CNNs via Learnable Agent Guidance and Alignment. Proceedings of the IEEE/CVF Conference on Computer Vision and Pattern Recognition (CVPR).

[20] Lin H., Bai H., Liu Z., Hou L., Sun M., Song L., Wei Y., Surr Z. MoPE-CLIP: Structured Pruning for Efficient Vision-Language Models with Module-wise Pruning Error Metric. Proceedings of the IEEE/CVF Conference on Computer Vision and Pattern Recognition (CVPR).

[21] Sabaghian M., Keyvanrad M.A., Moghadami S.M. (2025). A Novel Compression Framework for YOLOv8: Achieving Real-Time Aerial Object Detection on Edge Devices via Structured Pruning and Channel-Wise Distillation. arXiv.

[22] Lin Q., Yang X. (2025). KSG-YOLO: Application of YOLO-Based Detection with Knowledge Distillation and Structured Pruning in Green Assembly Building Scenarios. IEEE Access.

[23] Xu H., Li Y., Liu Y., Jiang Z. (2024). Structural Pruning for LiDAR 3D Detection Network by Dependency Graph. IET Conf. Proc..

[24] Huang H., Song H.-J., Zhao Q. (2025). Explainable Structured Pruning of BERT via Mutual Information. Entropy.

[25] Liu Y., Fan K., Wu D., Zhou W. (2023). Filter pruning by quantifying feature similarity and entropy of feature maps. Neurocomputing.

[26] Qiu Y., Niu L., Sha F., Cheng Z., Yanai K. (2025). Entropy-Guided Search Space Optimization for Efficient Neural Network Pruning. Algorithms.

[27] Lu Y., Guan Z., Yang Y., Zhao W., Gong M., Xu C. Entropy Induced Pruning Framework for Convolutional Neural Networks. Proceedings of the AAAI Conference on Artificial Intelligence (AAAI).

[28] Wang J., Tang A., Chen W., Zhang Y. An Enhanced YOLOv8 Network Combined with Model Pruning for Defect Detection in Solar Cell Images. Proceedings of the 2024 IEEE 8th International Conference on Vision, Image and Signal Processing (ICVISP).

[29] Muşat B., Andonie R. Accelerating Convolutional Neural Network Pruning via Spatial Aura Entropy. Proceedings of the 2023 27th International Conference Information Visualisation (IV).

[30] Ma J. (2025). Copula Entropy: Theory and Applications. arXiv.

[31] Ghosh I., Sunoj S. (2024). Copula-Based Mutual Information Measures and Mutual Entropy: A Brief Survey. Math. Methods Stat..

[32] Ma J. (2023). Two-Sample Test with Copula Entropy. arXiv.

[33] Rostami P., Sinha N., Chenni N., Kacem A., El Rahman Shabayek A., Shneider C., Aouada D. Information Theoretic Pruning of Coupled Channels in Deep Neural Networks. Proceedings of the IEEE/CVF Winter Conference on Applications of Computer Vision (WACV).

[34] Xu Y., Yang J., Wang R., Li H. (2024). An Effective Two-Stage Channel Pruning Method Based on Two-Dimensional Information Entropy. Appl. Intell..

[35] Cai Z., Chen R., Wu Z., Xue W. (2024). YOLOv8n-FAWL: Object Detection for Autonomous Driving Using YOLOv8 Network on Edge Devices. IEEE Access.

[36] Zhang H., Chan L.L.H. (2024). Overcoming the Challenges of Long-Tail Distribution in Nighttime Vehicle Detection. IEEE Intell. Syst..

[37] Zhao J., Li W., Zhu B., Zhang P., Hu Z., Meng J. (2025). Corner Case Dataset for Autonomous Vehicle Testing Based on Naturalistic Driving Data. Smart Cities.

[38] Wang Y., Huang W., Li J., Du G., Wang X., Wenjuan E., Shi J. (2024). A More Balanced Loss-Reweighting Method for Long-Tailed Traffic Sign Detection and Recognition. IEEE Trans. Intell. Transp. Syst..

[39] Ren N., Li X., Wu Y., Fu Y. (2024). Mixed Mutual Transfer for Long-Tailed Image Classification. Entropy.

[40] Kraskov A., Stögbauer H., Grassberger P. (2004). Estimating mutual information. Phys. Rev. E.

